# Molecular docking and molecular dynamics simulation study of inositol phosphorylceramide synthase – inhibitor complex in leishmaniasis: Insight into the structure based drug design

**DOI:** 10.12688/f1000research.9151.2

**Published:** 2016-09-01

**Authors:** Vineetha Mandlik, Shailza Singh

**Affiliations:** 1 National Centre for Cell Science, NCCS Complex, SP Pune University Campus, Pune, India

**Keywords:** IPCS (Inositol phosphorylceramide synthase), Sphingolipid metabolism, Leishmania, Leishmaniasis, Drug Inhibitor design, Coumarin derivatives, Molecular docking, Molecular dynamics simulation

## Abstract

Inositol phosphorylceramide synthase (IPCS) has emerged as an important, interesting and attractive target in the sphingolipid metabolism of *Leishmania. *IPCS catalyzes the conversion of ceramide to IPC which forms the most predominant sphingolipid in *Leishmania*. IPCS has no mammalian equivalent and also plays an important role in maintaining the infectivity and viability of the parasite. The present study explores the possibility of targeting IPCS; development of suitable inhibitors for the same would serve as a treatment strategy for the infectious disease leishmaniasis. Five coumarin derivatives were developed as inhibitors of IPCS protein. Molecular dynamics simulations of the complexes of IPCS with these inhibitors were performed which provided insights into the binding modes of the inhibitors. *In vitro* screening of the top three compounds has resulted in the identification of one of the compounds (compound 3) which shows little cytotoxic effects. This compound therefore represents a good starting point for further *in vivo* experimentation and could possibly serve as an important drug candidate for the treatment of leishmaniasis.

## Abbreviations

IPCS – Inositol phosphorylceramide synthase, IPC – Inositol phosphorylceramide, AUR1 – Aureobasidin 1, DAG – Diacylglycerol, RMSD – Root Mean Square Deviation, LINCS – Linear constraint solver, PME – Particle Mesh Ewald.

## Introduction

Leishmaniasis is a neglected tropical disease that is caused by the protozoan parasite *Leishmania*. Around 12 million people are affected by this disease worldwide. The mechanism of action of most of the anti-leishmanial compounds remains largely unknown. The first line treatment of cutaneous leishmaniasis involves the administration of antimony based compounds. Treatment of *L. major* amastigotes with Sb(V) has been found to induce apoptosis by the induction of oxidative-stress and increase in intracellular calcium ^1^. Non-antimony based treatments such as miltefosine, topical formulations of paromomycin are cost effective, convenient and less toxic than antimony based compounds. Amphotericin B being a liposomal formulation is expensive, has a low therapeutic index and is difficult to administer ^2^. Newer formulations for the treatment of this disease include the administration of miltefosine. Miltefosine (hexadecylphosphocholine), originally an anticancer drug has been reported to induce apoptosis of *L. major* amastigotes in the infected macrophages ^3^. However the growing problem of drug resistance to the existing chemotherapeutics as well as the quick adaptability of the parasite to the host immune responses has necessitated the development of newer treatment strategies for leishmaniasis ^4– 6^.

**Figure 1.**
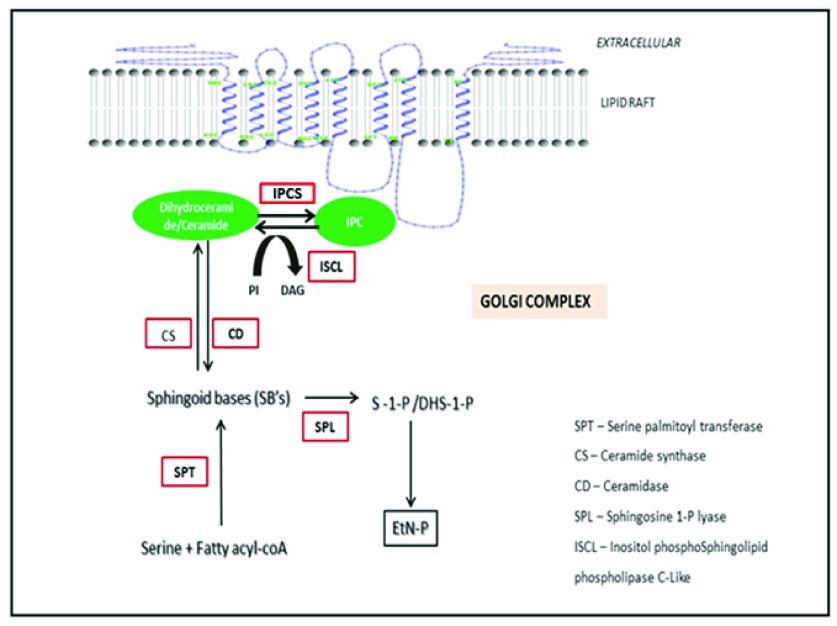
Role of IPCS in the sphingolipid metabolism of *Leishmania*. IPCS catalyzes the reaction involving the conversion of ceramide to IPC (Inositolphosphorylceramide). IPC forms the most predominant sphingolipid in *Leishmania*. IPCS plays an important role in maintaining the viability of the parasite.

Sphingolipids like IPC, form an important component of the parasitic membranes ^7^. IPCS (inositol phosphorylceramide synthase) is an enzyme involved in the sphingolipid metabolism of protozoans and other fungal species ^8^. The relative importance of IPCS in *Leishmania* has been identified through biochemical network modeling ^9^. IPCS catalyzes the conversion of ceramide to IPC which forms the most predominant sphingolipid of the parasite ^10^ ( Figure 1). IPCS also maintains the concentration of DAG and ceramide, both of which serve as secondary messengers in several signal transduction events ^11^. IPCS localizes into the lipid rafts of the Golgi complex ^12^. Lipid rafts have been proposed to involve in a wide array of events like trafficking of lipid modified proteins in addition to playing an important role in the formation of signal transduction complexes ^13^. IPCS has been important for maintaining the viability and the infectivity of several fungal species like *Cryptococcus neoformans*, *Candida albicans* and pathogens like *Leishmania* ^14– 17^. Interestingly there is no mammalian equivalent of this enzyme and the major sphingolipid in the host is sphingomyelin instead of IPC. Hence IPCS has been considered as a choke point enzyme in the sphingolipid metabolism of *Leishmania* thereby serving as a druggable target for the treatment of several fungal and protozoan diseases like leishmaniasis. *Lmj*IPCS comprises of 338 amino acids and has 6 transmembrane domains and belongs to the PAP2c family ^9^. IPCS is encoded by the AUR1 gene. IPCS protein present in fungi exhibits sensitivity to antifungal agents like galbonolide A, aureobasidin A, macrolidegalbonolide and khafrefungin ^18, 19^. IPCS has been recently discovered in *Leishmania* and to the best of our knowledge there are no reports of inhibitor design against this protein. This paper explores the possibility of targeting IPCS for the development of anti-protozoan compounds. An *in silico* approach for drug design has led to the development of five novel coumarin derivatives. The refinement and validation of the docked complexes has been done using molecular dynamics simulations to map the protein ligand interactions. Based on the *in silico* findings, the promising candidates were considered for further experimental evaluation and validation.

## Materials and methods

### Generation of the lead compounds

**Figure 2.**
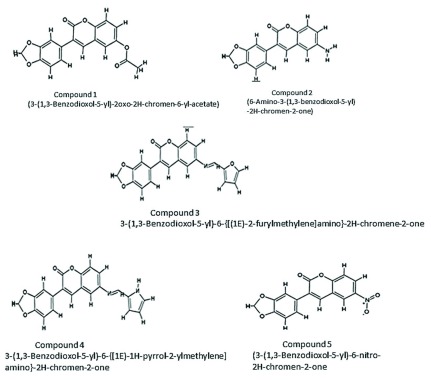
2D representation of the IPCS inhibitors. The designed inhibitors are Coumarin derivatives. Coumarin increases the phagocytic activity of the macrophages.

A set of coumarin derivatives were prepared by the assembly of pharmacophoric groups. The 2D structures of the inhibitors were drawn and edited using Chemsketch version 12.01 ^20^ ( Figure 2). The SMILES format for all the compounds was generated using Open Babel version 2.3.1 ^21^. Inhibitors were designed and filtered using the “Lipinski rules of five” ^22^ and Veber’s rules ^23^ using the Molinspiration Property Calculation Service ( www.molinspiration.com).

### Pharmacophore generation

The pharmacophore models describing the inherent chemical features of the inhibitors were generated using the “Feature mapping protocol” available in Discovery Studio version 3.0 ( www.accelyrs.com). Pharmacophore models of the inhibitors indicated that the ligand had at least a maximum of 5 pharmacophoric features i.e. Hydrogen bond acceptors (HBA), Hydrogen bond donors (HBD), positive ionizable groups (PI), Ring aromatic groups (RA) and the Hydrophobic groups (HY) present in the ligand.

### Molecular docking

IPCS is one of the emerging drug targets for the treatment of leishmaniasis. The crystal structure of the IPCS protein has not been solved and hence the 3D structure for the IPCS protein developed by our group before has been used for the inhibitor design. The model was developed using the I-TASSER server ( http://zhanglab.ccmb.med.umich.edu/I-TASSER/). The predicted model has a total of 338 amino acid residues and has 7 transmembrane helices ^9^. Binding site prediction of the IPCS protein was done using MetaPocket version 2.0 ^24^. The 3D structure of IPCS was energy minimized by the steepest gradient method of energy minimization using the GROMACS 4.0 package ^25^. Mol2 file format of the inhibitors was converted to PDBQT format using MGL tools prior to docking. All the water and solvent atoms of the protein were removed prior to docking and the polar hydrogens were added. The protein was kept rigid while the ligand was allowed to rotate and explore more flexible binding pockets. Docking of the inhibitors onto the IPCS protein was performed using Auto Dock 4 version 1.5.6 and Auto Dock vina version 1.1.2. The grid box was centered around the binding pocket. The grid dimensions were adjusted according to the binding site and the default scoring function was used for docking ^26, 27^. Binding modes of the docked complexes were obtained and the amino acid residues present at a distance of 5Å were considered as the binding partners of the ligands. The interaction diagrams representing the docked complexes have been generated using Pymol v 1.3.

### Molecular dynamics simulation of the docked complexes

Molecular dynamics simulation is a computational method that provides information regarding the time dependent behavior of any molecular system by integrating Newton’s laws of motion. The docked complexes (IPCS-inhibitor complex) were subjected to MD simulation using Desmond version 4.4 (Schodinger Biosuite). MD simulation of both the IPCS protein and IPCS –ligand complexes were performed for a time period of 10ns by using the OPLS force field. The complex was centered in a cubic box and filled with TIP3P water molecules. The system was neutralized and the initial energy minimization for the system was done using the conjugant gradient algorithm. The Martyna-Tobias-Klein scheme was used for pressure coupling. Electrostatic forces were calculated using the PME algorithm ^28^. All runs were performed at 300K at constant volume and temperature (NPT ensemble) under certain periodic boundary conditions. RMSD plots for the backbone atoms for both the protein and ligand bound protein were generated to understand the relative stability of the ligand inside its binding pocket and the IPCS-inhibitor complexes were visualized.

### Flow cytometry

Macrophage cell population was collected post 24 h treatment with the compound 3, washed and suspended in 1XPBS. Cells were stained with 10µl of 10μg/mL of propidium iodide (PI) dye (Invitrogen) and acquired on FACS. Total macrophage population was gated based on their forward scatter (FSC) and side scatter (SSC) after excluding the cell debris. A minimum of 10,000 events were acquired for each sample on FACS Canto II (Beckon Dickson, San Jose, California) and analyzed using FACS Diva Software (version 6.2.1) (Beckon Dikson, San Jose, California).

## Results

Raw data for ‘Molecular docking and molecular dynamics simulation study of inositol phosphorylceramide synthase – inhibitor complex in leishmaniasis: Insight into the structure based drug design’ A description of each file is provided in ‘Dataset descriptions’. Click here for additional data file. Copyright: © 2016 Mandlik V and Singh S 2016 Data associated with the article are available under the terms of the Creative Commons Zero "No rights reserved" data waiver (CC0 1.0 Public domain dedication).

A group of coumarin derivatives were prepared as inhibitors of the IPCS protein belonging to *L. major*. Assessment of the drug like properties indicated that all the inhibitors were found to comply with the Lipinski’s “Rule of five” (molecular weight ( *M*_wt_) ≤ 500, clogP ≤ 5, H-bond donors (HBD) ≤ 5, and acceptors (HBA) ≤ 10) and Verber’s rules (no. of rotatable bonds < 10, PSA ≤ 140A ^2^) ( Table 1).

**Table 1.** Molecular descriptors of the lead compounds. HBA – Hydrogen bond acceptor, HBD – Hydrogen bond donor, HY – Hydrophobic, RA – Ring aromatic, MR – Molar refractivity, NROTB – No. of rotatable bonds, cLog *P* – log octanol/water partition coefficient, PSA – Polar surface area, NSC – No. of stereo centers.

S.No	Mwt	cLog *P*	HBA	HBD	HY	RA	MR	NROTB	PSA(A ^2^)	NSC
1	324	2.8	8	0	2	4	83.82	3	74.98	0
2	281	2.46	5	2	2	4	76.92	1	74.70	0
3	359	4.22	7	0	3	6	78.47	4	73.84	1
4	358	3.86	6	1	3	6	100.70	1	77.36	1
5	311	3.15	9	0	2	4	78.65	2	94.50	0

### Molecular docking

**Figure 3.**
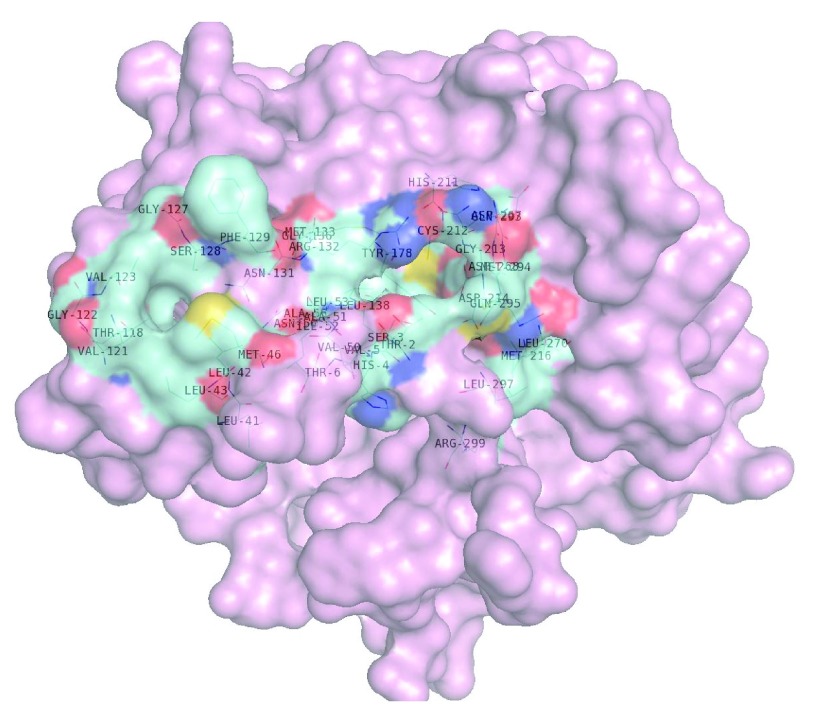
Binding site of the IPCS protein as predicted by Metapocket has been represented with carbon atoms in green, sulfur in yellow, nitrogen in blue and oxygen in red.

**Figure 4.**
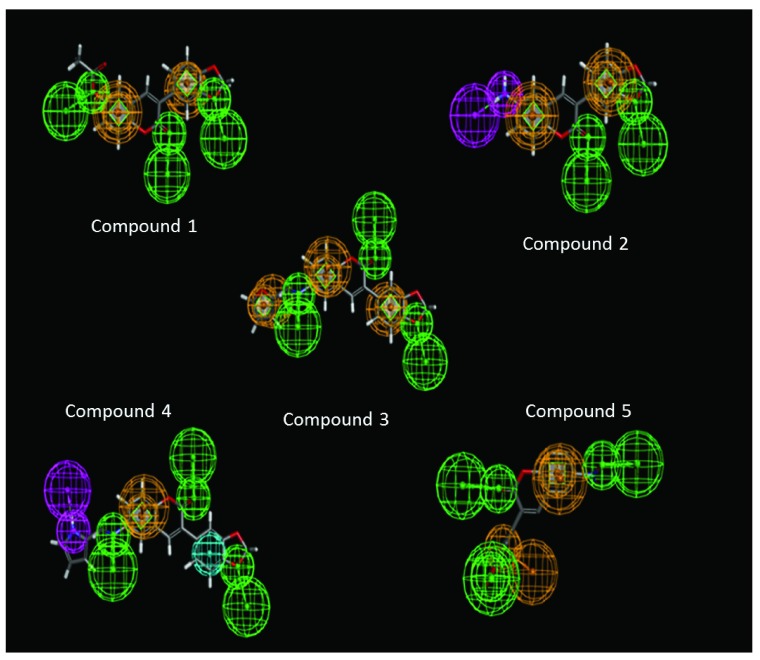
Pharmacophore models of the inhibitors. The pharmacophoric features such as hydrogen bond acceptors (green), hydrogen bond donors (pink), hydrophobic regions (blue) and the aromatic rings in yellow are shown in the figure.

Binding site of the IPCS protein [ Figure 3], the structure was refined using molecular dynamics simulation. Molecular docking studies reveal the binding modes of the ligand with IPCS protein giving an insight into the crucial amino acid residues that are involved during the binding. A comparison of the binding energies of all the compounds indicates that compound 3 has the least binding energy among all and hence exhibits maximum affinity towards the IPCS protein ( Table 2). The interaction modes of all the IPCS inhibitors post docking along with their pharmacophoric features have been presented [ Figure 4]. Binding mode analysis reveals that hydrophilic amino acids like Arg299 and His220 were found to be involved in hydrogen or π bonding with most of the ligands ( Table 3). The relative stability of the compounds within the binding site was maintained due to the van der Waal’s interaction between the hydrophobic amino acids of the IPCS protein and the ligand ( Table 4).

**Table 2.** The approximate free energies of binding (ΔG _b_) of the compounds calculated by Autodock vina.

S.No	Compound Name	Binding energy (Kcal/mol)
1.	(3-(1,3-Benzodioxol-5-yl)-2oxo-2H-chromen-6-yl-acetate)	-9.0
2.	(6-Amino-3-(1,3-benzodioxol-5-yl)-2H-chromen-2-one)	-8.4
3.	3-(1,3-Benzodioxol-5-yl)-6-{[(1E)-2- furylmethylene]amino}-2H-chromen-2-one	-9.8
4.	3-(1,3-Benzodioxol-5-yl)-6-{[(1E)-1H-pyrrol-2- ylmethylene]amino}-2H-chromene-2-one	-9.5
5.	(3-(1,3-Benzodioxol-5-yl)-6-nitro-2H-chromen-2-one	-9.0

**Table 3.** IPCS –inhibitor interactions post docking.

Compound	Amino acid	Ligand	Type of interaction
1	His220	1,3 benzodioxol group	Hydrogen bonding
1	Asn183	1,3 benzodioxol group	Hydrogen bonding
3	Phe129	1,3 benzodioxol group	Sigma bond formation
4	Arg299	1,3 benzodioxol group	Hydrogen bonding
5	His220	1,3 benzodioxol group	Hydrogen bonding
5	Glu192	Chromene group	Sigma bond formation
5	Arg299	Chromene group	Pi bonding

**Table 4.** Comparison of the interacting residues both pre and post MD simulation.

Compound	Binding interactions post docking (pre MD simulation)	Binding interactions post MD simulation
1.	Ile223, Met222, Asn183, Asp182, Pro252, Tyr255, Val195, Pro188, Glu192, Leu196	Tyr256, Prot188, Glue192, Tyr255, Leu196
2.	Arg299, Asp214, Thr6, Ala7, Leu138, Thr323, Ala325, Asp61, Met59, Pro62, Ala57	Arg299, Met59, Ala57, Leu138, Ala7, Pro62, Asp61, Thr323
3.	Arg132, Ala51, Leu130, Val172, Gly49, Met46, Val150	Met46, Phe129, Asn131, Arg132
4.	Pro62, Tyr178, Asp214, Thr6, Ala7, Trp23, Asp19, Ile298, Val5, Leu138, Thr323	Glu63, Val321, Gln322, Arg299, Asp61, Val5, Ile298, Leu270, Ala55, Leu138, Pro62, Met59, Thr323, Asp19, Val10
5.	Tyr255, Asp182, Pro252, Asn187, Gln189	Ile256, Leu259, Leu196, Ile199, Glu192, Asn187, Tyr256, Tyr255

### Molecular dynamics simulation of the docked complexes

**Figure 5.**
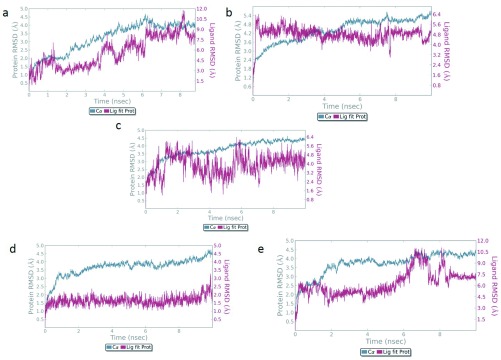
RMSD of the IPCS-ligand complexes. Backbone RMSD of **a**) Compound 1 and **b**) Compound 2 **c**) Compound 3 **d**) Compound 4 **e**) Compound 5 is shown in the figure. Compound 1, 2 and 3 appear to maintain their stability within the binding pocket as they show lower RMSD fluctuations.

**Figure 6a–e.**
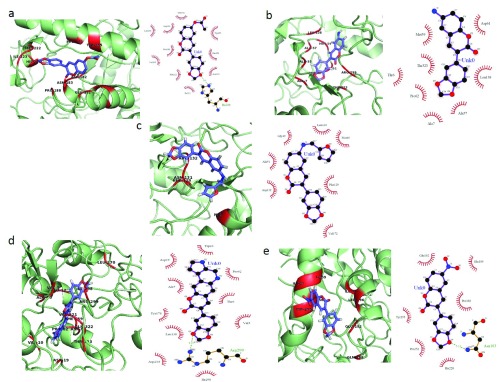
Binding modes of IPCS-ligand complexes. The interaction of the ligand within the IPCS inhibitor complex post MD simulation is shown the figure **a**) IPCS - compound 1 complex **b**) IPCS - compound 2 complex **c**) IPCS - compound 3 complex **d**) IPCS - compound 4 complex and **e**) IPCS - compound 5 complex. MD simulation was performed for a time period of 10ns. Interacting residues are represented in red.

Protein backbone RMSD plots indicate the stability of the IPCS-inhibitor complex. The drug backbone RMSD plots indicate that compounds 2 and 3 maintained their interactions with the IPCS protein ( Figure 5). Binding modes of compounds 1 to 5 post MD simulation have been shown in Figure 6a–e.

### Cytotoxicity of the proposed inhibitors

**Figure 7.**
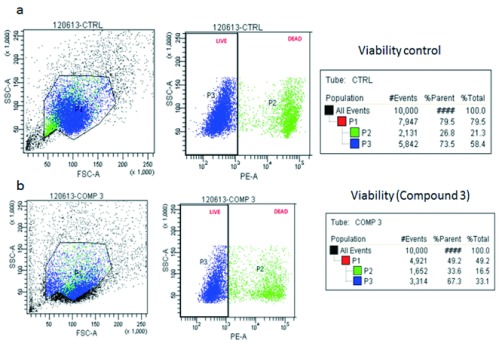
FACS analysis for measuring macrophage cell viability. Macrophages were treated with compound 3 for 24h. **a**) Control cell population displayed a percentage viability of 73.5% **b**) Compound 3 (1mg/ml) treated macrophages displayed a viability of 67.3% post 24hr treatment.

The cytotoxicity profile of compound 3 was checked over the macrophage cell line. Of all five compounds, compound 3 had the highest viability. The viability of C3 treated macrophages (67.3%) was slightly lesser than the control (73.5%) ( Figure 7).

## Discussion

IPCS (Inositol phosphorylceramide synthase) has been identified as an important drug target in the sphingolipid metabolism of several organisms like fungi, yeast and protozoans like *Leishmania* and *Trypanosoma* ^29^. Systems biology has played a major role in defining the relative importance of IPCS in the sphingolipid metabolism of *Leishmania*, a protozoan responsible for causing an infectious disease leishmaniasis. The quest for developing new inhibitors for any target protein relies mainly on *in silico* approaches like computer based docking which involves the generation of a comprehensive set of ligand conformations that are eventually scored and ranked according to their stability and affinity for the protein. Coumarin has been shown to simulate the macrophages, enhancing their phagocytic ability ^30^. A total of five ligands were developed as inhibitors for the IPCS protein. Molecular docking of the inhibitors with the IPCS protein revealed the binding modes of inhibitors. To account for the flexibility of the protein and ligand and to determine the binding affinity of the inhibitors with the IPCS protein, a 10 ns molecular dynamics simulation of the docked complexes was carried out. Binding mode analysis revealed that the binding modes obtained after MD simulation were more or less similar to that obtained post docking ( Table 4). The presence of a large number of H bond acceptors, H bond donors as well as hydrophobic groups in the ligands account for the stability of the ligand inside the binding pocket of IPCS. Based on the RMSD of the ligand-protein complex, it was observed that compounds 1, 2 and 3 maintained their interaction with the protein with lower RMSD fluctuations. Out of these, compound 3 showed the highest binding affinity and its cytotoxicity was assessed using flow cytometry. Cytotoxicity of compound 3 was lesser as compared to other compound. A comparison of the compound 3 treated macrophages along with the untreated macrophages has been made in Figure 6.

## Conclusion

There is an urgent need to design and develop novel anti-leishmanial compounds due to various problems associated with the current chemotherapeutics for the treatment of this disease. IPCS has been proposed to be a probable drug target in the sphingolipid pathway of *Leishmania*. We have designed a few novel coumarin derivatives using *in silico* approaches. MD simulation post docking studies reveal the interactions between the IPCS protein and ligands. Binding modes obtained after docking and after MD simulation reveal almost identical binding modes which is suggestive of the selectivity and selectivity of the ligand towards the active site of the IPCS protein.

## Data availability

The data referenced by this article are under copyright with the following copyright statement: Copyright: © 2016 Mandlik V and Singh S

Data associated with the article are available under the terms of the Creative Commons Zero "No rights reserved" data waiver (CC0 1.0 Public domain dedication). 

*F1000Research*: Dataset 1. Raw data for ‘Molecular docking and molecular dynamics simulation study of inositol phosphorylceramide synthase – inhibitor complex in leishmaniasis: Insight into the structure based drug design’, 10.5256/f1000research.9151.d128337 ^31^

## References

[1] Haldar AK Sen P Roy S: Use of antimony in the treatment of leishmaniasis: current status and future directions. *Mol Biol Int.* 2011;2011:23, 571242. 10.4061/2011/571242 22091408 PMC3196053

[2] Yardley V Croft SL: Activity of liposomal amphotericin B against experimental cutaneous leishmaniasis. *Antimicrob Agents Chemother.* 1997;41(4):752–756. 9087483 10.1128/aac.41.4.752 PMC163788

[3] Khademvatan S Gharavi MJ Rahim F: Miltefosine-induced apoptotic cell death on *Leishmania major* and *L. tropica* strains. *Korean J Parasitol.* 2011;49(1):17–23. 10.3347/kjp.2011.49.1.17 21461264 PMC3063921

[4] Hadighi R Mohebali M Boucher P: Unresponsiveness to Glucantime treatment in Iranian cutaneous leishmaniasis due to drug-resistant *Leishmania tropica* parasites. *PLoS Med.* 2006;3(5):e162. 10.1371/journal.pmed.0030162 16605301 PMC1435779

[5] Mishra BB Kale RR Singh RK: Alkaloids: future prospective to combat leishmaniasis. *Fitoterapia.* 2009;80(2):81–90. 10.1016/j.fitote.2008.10.009 19015012

[6] Croft SL Barrett MP Urbina JA: Chemotherapy of trypanosomiases and leishmaniasis. *Trends Parasitol.* 2005;21(11):508–512. 10.1016/j.pt.2005.08.026 16150644

[7] Heung LJ Luberto C Del Poeta M: Role of sphingolipids in microbial pathogenesis. *Infect Immun.* 2006;74(1):28–39. 10.1128/IAI.74.1.28-39.2006 16368954 PMC1346627

[8] Lester RL Dickson RC: Sphingolipids with inositolphosphate-containing head groups. *Adv Lipid Res.* 1993;26:253–274. 8379454

[9] Mandlik V Shinde S Chaudhary A: Biological network modeling identifies IPCS in *Leishmania* as a therapeutic target. *Integr Biol (Camb).* 2012;4(9):1130–1142. 10.1039/C2IB20037F 22842708

[10] Dickson RC: Sphingolipid functions in *Saccharomyces cerevisiae*: comparison to mammals. *Annu Rev Biochem.* 1998;67:27–48. 10.1146/annurev.biochem.67.1.27 9759481

[11] Cerbón J Falcon A Hernández-Luna C: Inositol phosphoceramide synthase is a regulator of intracellular levels of diacylglycerol and ceramide during the G _1_ to S transition in *Saccharomyces cerevisiae*. *Biochem J.* 2005;388(Pt 1):169–176. 10.1042/BJ20040475 15560753 PMC1186705

[12] Denny PW Shams-Eldin H Price HP: The protozoan inositol phosphorylceramide synthase: a novel drug target that defines a new class of sphingolipid synthase. *J Biol Chem.* 2006;281(38):28200–28209. 10.1074/jbc.M600796200 16861742 PMC1817671

[13] Simons K Sampaio JL: Membrane organization and lipid rafts. *Cold Spring Harb Perspect Biol.* 2011;3(10):a004697. 10.1101/cshperspect.a004697 21628426 PMC3179338

[14] Henry J Guillotte A Luberto C: Characterization of inositol phospho-sphingolipid-phospholipase C 1 (Isc1) in *Cryptococcus neoformans* reveals unique biochemical features. *FEBS Lett.* 2011;585(4):635–640. 10.1016/j.febslet.2011.01.015 21256847 PMC3045780

[15] Wells GB Dickson RC Lester RL: Isolation and composition of inositolphosphorylceramide-type sphingolipids of hyphal forms of *Candida albicans*. *J Bacteriol.* 1996;178(21):6223–6226. 8892822 10.1128/jb.178.21.6223-6226.1996 PMC178493

[16] Levine TP Wiggins CA Munro S: Inositol phosphorylceramide synthase is located in the Golgi apparatus of *Saccharomyces cerevisiae*. *Mol Biol Cell.* 2000;11(7):2267–2281. 10.1091/mbc.11.7.2267 10888667 PMC14918

[17] Güther ML Lee S Tetley L: GPI-anchored proteins and free GPI glycolipids of procyclic form *Trypanosoma brucei* are nonessential for growth, are required for colonization of the tsetse fly, and are not the only components of the surface coat. *Mol Biol Cell.* 2006;17(12):5265–5274. 10.1091/mbc.E06-08-0702 17035628 PMC1679689

[18] Nagiec MM Nagiec EE Baltisberger JA: Sphingolipid synthesis as a target for antifungal drugs. Complementation of the inositol phosphorylceramide synthase defect in a mutant strain of *Saccharomyces cerevisiae* by the *AUR1* gene. *J Biol Chem.* 1997;272(15):9809–9817. 10.1074/jbc.272.15.9809 9092515

[19] Yano T Aoyagi A Kozuma S: Pleofungins, novel inositol phosphorylceramide synthase inhibitors, from *Phoma* sp. SANK 13899. I. Taxonomy, fermentation, isolation, and biological activities. *J Antibiot (Tokyo).* 2007;60:136–142. 10.1038/ja.2007.13 17420564

[20] ACD/Chemsketch, version 5.12. Advanced Chemistry Development, Inc., Toronto, ON, Canada. Reference Source

[21] O'Boyle NM Banck M James CA: Open Babel: An open chemical toolbox. *J Cheminform.* 2011;3:33. 10.1186/1758-2946-3-33 21982300 PMC3198950

[22] Lipinski CA Lombardo F Dominy BW: Experimental and computational approaches to estimate solubility and permeability in drug discovery and development settings. *Adv Drug Deliv Rev.* 1997;23(1-3):3–25. 10.1016/S0169-409X(96)00423-1 11259830

[23] Veber DF Johnson SR Cheng HY: Molecular properties that Influence the oral bioavailability of drug candidates. *J Med Chem.* 2002;45(12):2615–2623. 10.1021/jm020017n 12036371

[24] Huang B: MetaPocket: a meta approach to improve protein ligand binding site prediction. *OMICS.* 2009;13(4):325–330. 10.1089/omi.2009.0045 19645590

[25] Berendsen HJ van der Spoel D van Drunen R: GROMACS: a message-passing parallel molecular dynamics implementation. *Comp Phys Comm.* 1995;91(1–3):43–56. 10.1016/0010-4655(95)00042-e

[26] Morris GM Goodsell DS Halliday RS: Automated docking using a Lamarckian genetic algorithm and an empirical binding free energy function. *J Computational Chemistry.* 1998;19(14):1639–1662. 10.1002/(SICI)1096-987X(19981115)19:14<1639::AID-JCC10>3.0.CO;2-B

[27] Trott O Olson AJ: AutoDock Vina: improving the speed and accuracy of docking with a new scoring function, efficient optimization, and multithreading. *J Comput Chem.* 2010;31(2):455–461. 10.1002/jcc.21334 19499576 PMC3041641

[28] Darden T York D Pedersen L: Particle mesh Ewald: an *N*-log( *N*) method for Ewald sums in large systems. *J Chem Phys.* 1993;98(12):10089–10092. 10.1063/1.464397

[29] Zhang K Beverley SM: Phospholipid and sphingolipid metabolism in *Leishmania*. *Mol Biochem Parasitol.* 2010;170(2):55–64. 10.1016/j.molbiopara.2009.12.004 20026359 PMC2815228

[30] Piller NB: A morphological assessment of the stimulatory effect of coumarin on macrophages. *Br J Exp Pathol.* 1978;59(1):93–96. 638035 PMC2041321

[31] Mandlik V Singh S: Dataset 1 in: Molecular docking and molecular dynamics simulation study of inositol phosphorylceramide synthase – inhibitor complex in leishmaniasis: Insight into the structure based drug design. *F1000Research.* 2016 Data Source 10.12688/f1000research.9151.1 PMC5089144 27853511

