# Supplementary material for: Molecular docking and molecular dynamics simulation study of inositol phosphorylceramide synthase – inhibitor complex in leishmaniasis: Insight into the structure based drug design
Source: F1000Res. 2016 Sep 1;5:1610. Originally published 2016 Jul 8. [Version 2] doi: 10.12688/f1000research.9151.2 (PMC5089144; doi:10.12688/f1000research.9151.2)
Supplement: Raw data for ‘Molecular docking and molecular dynamics simulation study of inositol phosphorylceramide synthase – inhibitor complex in leishmaniasis: Insight into the structure based drug design’ — A description of each file is provided in ‘Dataset descriptions’. [file f1000research-5-10109-s0000.tgz › 3c18566b-4baa-4f04-b71e-a56099fd744a_Raw_data.zip!/Description of datasets.docx]

The dataset includes

a) Model of IPCS protein built using ITASSER server.
b) Dataset 1: Mol file of the compound 1, PDB structure of the docked complex 1, Minimized structure post MD simulation of complex 1.
c) Dataset 2: Mol file of the compound 2, PDB structure of the docked complex 1, Minimized structure post MD simulation of complex 2.
d) Dataset 3: Mol file of the compound 3, PDB structure of the docked complex 3, Minimized structure post MD simulation of complex 3.
e) Dataset 4: Mol file of the compound 4, PDB structure of the docked complex 4, Minimized structure post MD simulation of complex 4.
f) Dataset 5: Mol file of the compound 5, PDB structure of the docked complex 5, Minimized structure post MD simulation of complex 5.
