# Supplementary material for: Molecular docking and molecular dynamics simulation study of inositol phosphorylceramide synthase – inhibitor complex in leishmaniasis: Insight into the structure based drug design
Source: F1000Res. 2016 Sep 1;5:1610. Originally published 2016 Jul 8. [Version 2] doi: 10.12688/f1000research.9151.2 (PMC5089144; doi:10.12688/f1000research.9151.2)
Supplement: Peer review history

# Peer review: Referee response for version 2

**DOI:** 10.5256/f1000research.10109.r17089

**Published:** 2016-10-25

**Authors:** Vigneshwaran Namasivayam

**Affiliations:** 1 PharmaCenter Bonn, Pharmaceutical Institute, Pharmaceutical Chemistry I, University of Bonn, Bonn, Germany

Mandlik and Singh have applied the pharmcophore model and physicochemical properties to select five coumarin derivatives as a starting point as inhibitors targeting inositol phosphorylceramide synthase a target selected to study sphingolipid metabolism of Leishmania. IPCS is an important target because there is no mammalian equivalent of this enzyme. The authors have utilized the state-of-the art computational techniques molecular docking and molecular dynamics to confirm the binding mode and its interaction with the enzyme. The analysis explain that the high number of H-bond acceptors, donors and hydrophobic groups introduce strong interactions of the ligand within the binding site of the enzyme. The cytotoxicity profile of the selected inhibitors provides additional information about the coumarin derivatives. Thus, this study is a good starting point for focusing coumarin derivatives as inhibitors targeting against Leishmania. In the future study, it can be extended to modify these molecules to further enhance the interaction with IPCS and other experimental studies to confirm the binding affinity.

I have read this submission. I believe that I have an appropriate level of expertise to confirm that it is of an acceptable scientific standard.

# Peer review: Referee response for version 2

**DOI:** 10.5256/f1000research.10109.r16005

**Published:** 2016-09-26

**Authors:** François Ferron

**Affiliations:** 1 UMR 7257, Architecture and Functions of Biological Macromolecules Laboratory, CNRS-Aix-Marseille University, Marseille, France

Authors have answered the Referee comments and improved the paper enough to be indexed.

I have read this submission. I believe that I have an appropriate level of expertise to confirm that it is of an acceptable scientific standard.

# Peer review: Referee response for version 1

**DOI:** 10.5256/f1000research.9848.r14858

**Published:** 2016-07-20

**Authors:** François Ferron

**Affiliations:** 1 UMR 7257, Architecture and Functions of Biological Macromolecules Laboratory, CNRS-Aix-Marseille University, Marseille, France

Mandlik and Singh are presenting a very interesting study against *Leishmania.* Using *in silico* methods they have identified promising compounds.

 They chose with great reason IPCS as a target for its uniqueness to the pathogens the study very interesting yet the manuscript needs some clarifications.

1. As there is no structure the authors have done a model, the model should be presented here and I feel a previous reference from 2012 won't do. The reader needs to be introduced to it and at least to have a clear understanding of the catalytic site and docking site.
2. In the material and methods it would be appreciated to have an idea where is the center of the grid (may be with a figure).
3. Can you explain why you chose a grid with large dimensions?
4. The analysis of the viability raises a question. Why the surface p1 is not the same between control and compound? To compare the stats it should be the same size here it is half. Also from fig 6 compound 3 it seems that a lot of data were excluded from P1 area? Can you explain?
5. As perspective are you planning on testing *in vitro* the efficiency of compound 3 and have an idea of binding affinity?

Minor comment on figure 2: structures of compounds are distorted and could you put all the compound in same orientation 1 (and 2 are upside down). It will help to compare the geometry and differences between molecules.

 Figure 5: description of interactions could be better  represented ma be with LigPlot, as it is it is not clear.

I have read this submission. I believe that I have an appropriate level of expertise to confirm that it is of an acceptable scientific standard, however I have significant reservations, as outlined above.

# Response

**Published:** 2016-08-01

**Authors:** Shailza Singh

**Affiliations:** National Centre for Cell Science, India

1. *As there is no structure the authors have done a model, the model should be presented here and I feel a previous reference from 2012 won't do. The reader needs to be introduced to it and at least to have a clear understanding of the catalytic site and docking site.*
  Author’s response: The authors have accepted the suggestion made and have now included a figure showing the binding cavity that was predicted for the IPCS protein around which the grid box was centered during docking.
2. *In the material and methods it would be appreciated to have an idea where the center of the grid is (may be with a figure).*
  Author’s response: Figure 3 has been included keeping in mind the suggestion made.
3. *Can you explain why you chose a grid with large dimensions?*
  Author’s response: The inhibitors designed have not yet been reported. As there are no studies indicating the exact binding site in the IPCS protein, we have made binding site prediction and the grid box dimensions have been adjusted to incorporate most of the amino acids that fall in the binding site.
4. *The analysis of the viability raises a question. Why the surface p1 is not the same between control and compound? To compare the stats it should be the same size here it is half. Also from fig 6 compound 3 it seems that a lot of data were excluded from P1 area? Can you explain?*
  Author’s response:  The P1 area has been demarcated as per the untreated macrophages (control). As was a decrease in granularity of macrophages post treatment, the cells had lower SSC, however the viability of the cells has not decreased. As per the author’s knowledge about flow cytometry, the P1 area has to remain the same both for the control and treated samples.
5. *As perspective are you planning on testing in vitro the efficiency of compound 3 and have an idea of binding affinity?*
  Author’s response:  At present, the authors don’t have idea of the binding affinity of compound 3. Such studies can be done in future.
6. *Minor comment on figure 2: structures of compounds are distorted and could you put all the compound in same orientation 1 (and 2 are upside down). It will help to compare the geometry and differences between molecules.*
  Author’s response:  Changes suggested have been made in the Figure 2.
7. * Figure 5: description of interactions could be better represented maybe with LigPlot, as it is it is not clear.*
  Author’s response:  Changes suggested have been made in the Figure 6.

We thank the reviewer for his valuable suggestions which went a long way in improving the said manuscript.
